# Sarcomatoid Urothelial Carcinoma of the Urinary Bladder With Chondrosarcomatous and Concurrent Divergent Squamous Cell Carcinoma Differentiation– A Rare Entity

**DOI:** 10.7759/cureus.33107

**Published:** 2022-12-29

**Authors:** Atanu Kumar Pal, Bheemanathi Hanuman Srinivas, Sidhartha Kalra, Lalgudi N Dorairajan, Sreerag Sreenivasan Kodakkattil

**Affiliations:** 1 Urology, Jawaharlal Institute of Postgraduate Medical Education and Research, Puducherry, IND; 2 Pathology, Jawaharlal Institute of Postgraduate Medical Education and Research, Puducherry, IND

**Keywords:** urinary bladder, squamous cell carcinoma, sarcomatoid urothelial carcinoma, malignancy, chondrosarcoma

## Abstract

We present an extremely rare case where the sarcomatoid urothelial carcinoma of the urinary bladder was present with chondrosarcomatous and squamous cell differentiation.

A 74-year-old male smoker presented with intermittent hematuria with the passage of clots. On imaging, an irregular polypoidal lesion was diagnosed near the right vesicoureteric junction, and transurethral resection of the bladder tumor was performed. Histopathological examination showed sarcomatoid urothelial carcinoma with chondrosarcoma and squamous cell differentiation. He refused the surgical treatment of radical cystectomy and underwent Gemcitabine and Cisplatin chemotherapy. He died nine months after the diagnosis. Sarcomatoid urothelial carcinoma is a high-grade biphasic neoplasm with malignant epithelial and mesenchymal components. Its association with squamous cell carcinoma is infrequent. It is very aggressive, and there is no standard treatment for this disease. The radical surgical option appears to be the main form of treatment. It is scarce with a grave prognosis.

## Introduction

In 1856, Ordonez recorded the first report of a malignant bladder tumor containing elements of cartilage and bone [1]. Sarcomatoid urothelial carcinoma of the bladder is an unusual malignancy. It varies in histology, with the most common elements including high-grade spindle or pleomorphic cells, leiomyosarcoma, and malignant fibrous histiocytoma, osteo, and chondrosarcoma [2]. Chondrosarcoma is expected in the pelvis and femur but extremely rare in the urinary bladder, and its admixture with squamous cell carcinoma is even rarer [3]. In our case, the simultaneous presence of squamous cell carcinoma and chondrosarcoma makes it a rare case in the literature.

## Case presentation

**Figure 1:**
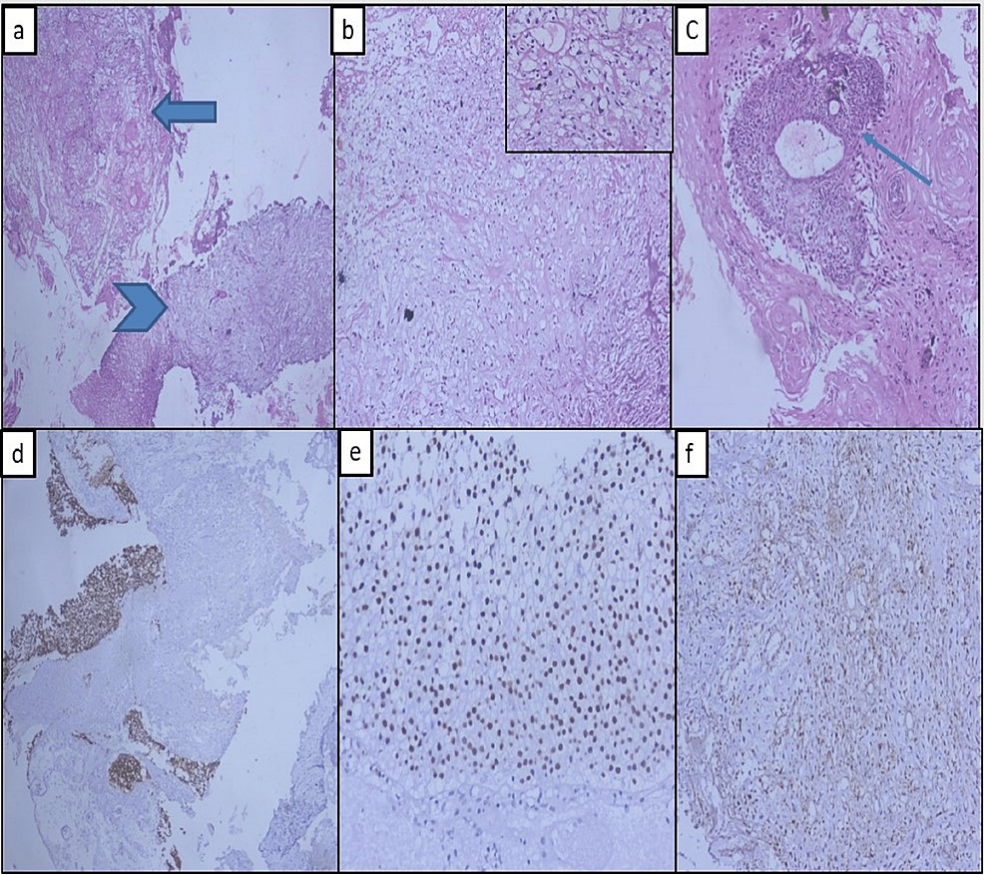
Histopathological images of the lesion after TURBT (Transurethral resection of bladder tumor) a) both squamous(arrow) and chondroid(arrowhead) elements (H&EX40); b) sheets of chondrocytes (inset shows atypical chondrocytes in high power) (H&EX200); c) squamous pearls admixed with central urothelial cells (line arrow) (H&EX100); d & e) Immunohistochemistry with CK7&GATA 3 show positivity in overlying malignant epithelial cells (DABX40&DABX200); f) IHC with S100 show positivity in chondrocytes (DABX200).

**Figure 2:**
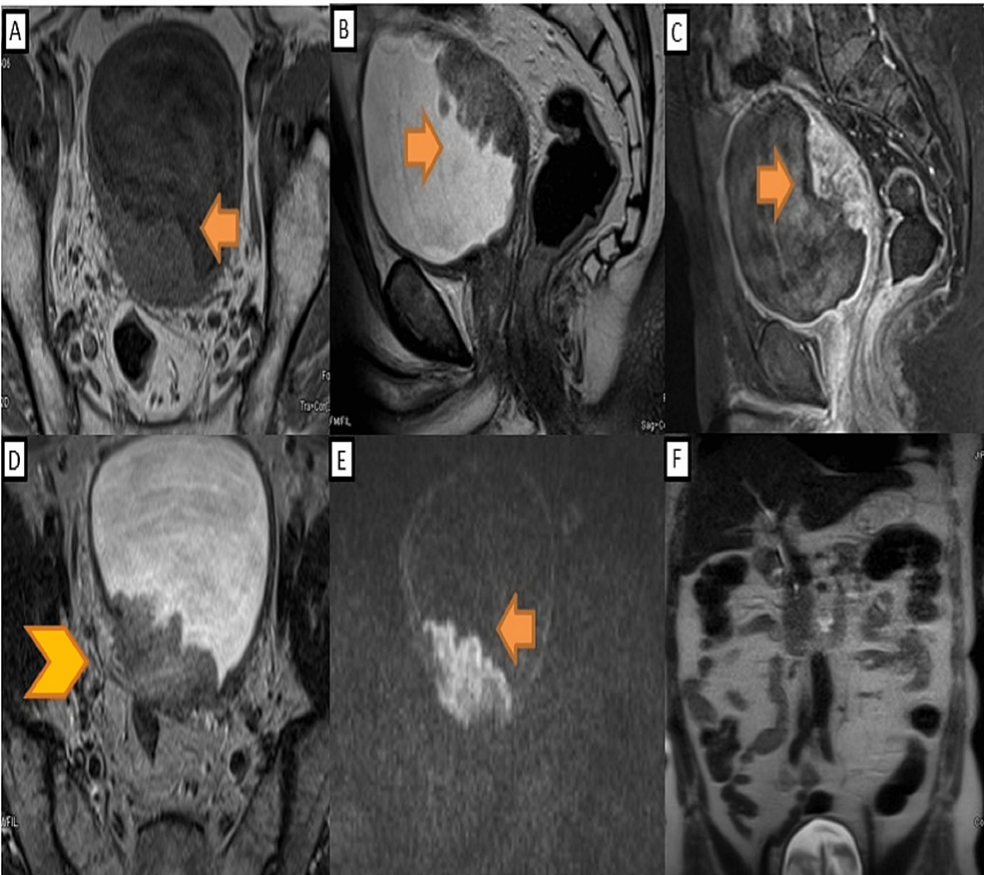
MRI (Magnetic Resonance Imaging) of the lesion A) T1 axial section shows an ill-defined irregular isointense mass lesion of 5x3.5x2.5 cm on the right lateral and posterior wall of the bladder (arrow); B) T2 sagittal section shows hypo to the intermediate signal intensity of the mass lesion (arrow); C) T1 post-contrast fat-suppressed image shows heterogeneous enhancement of the lesion (arrow); D) T2 axial section shows peri-vesical fat invasion and loss of fat plane with the anterior wall of the rectum (arrowhead); E) Diffusion Weighted image shows the urinary bladder mass (arrow); F) T2 HASTE (Half Fourier Single-shot Turbo spin-Echo) shows no lymph node or solid organ metastasis. The lesion shows VIRADS 5 (Vesical Imaging-Reporting and Data System).

A 74-year-old patient presented to our Institution with complaints of intermittent hematuria for the last three months with the passage of lump-like clots. Past medical history was unremarkable except for smoking for 30 years (60 pack-years). His Eastern Cooperative Oncology Group (ECOG) score was zero. He was clinically stable with no sign of malnutrition. No mass was palpable on per abdominal and digital rectal examinations. On blood investigations, her hemoglobin, total leukocyte count (TLC), platelet, urea, creatinine, sodium, potassium, calcium, and serum albumin were 8.2 g/dl, 9.85/μl, 1.75 lakhs, 23 mg/dl, 0.95 mg/dl, 134 meq/l, 4.4 meq/l, 8.7mg/dl, and 3.1 g/dl respectively. His liver function tests were within normal limits. Urine cytology was positive for high-grade malignancy. The urine culture was sterile. Ultrasound showed an irregular lobulated mass lesion of 5x3.5 cm in the right lateral wall. Computed tomography (CT) scan with Urography showed an irregular nodular broad-based polypoidal lesion along the right lateral wall of the urinary bladder near the right vesicoureteric (VU) junction with no hydroureteronephrosis. Transurethral resection of bladder tumor (TURBT) was performed, which revealed a large irregular polypoidal lesion near the right VU junction in the right lateral wall of the bladder. Complete resection was performed, and a deep muscle biopsy was taken. The histopathological examination was sarcomatoid urothelial carcinoma with heterogeneous elements like chondrosarcoma and squamous cell carcinoma with lamina propria and muscularis propria infiltration. The epithelial elements are positive for GATA 3 and CK7, and chondrosarcomatous elements are positive for S100 (Figure 1). Staging work-up with CT chest and abdomen showed no metastatic lesion in the lungs and liver. MRI of the pelvis also revealed an ill-defined irregular T1 iso/T2 hypo to intermediate signal intensity mass lesion right lateral and posterior wall of the bladder with diffusion restriction and heterogenous enhancement showing perivesical fat invasion and focal loss of fat planes with rectum posteriorly (Figure 2). It came as VI-RADS 5 (Vesical Imaging-Reporting and Data System). The TNM (tumor-T, nodes-N, and metastases-M) staging of the tumor was T3bN0M0 [4].

After discussion in the institutional tumor board meeting, he was given the surgical option of radical cystoprostatectomy with pelvic lymph node dissection with ileal conduit diversion. He was counseled about the advantages and disadvantages of all available modalities. His family members were also involved in the discussion.

He refused any form of surgical management because of the procedure's invasiveness. He took three cycles of Gemcitabine and Cisplatin chemotherapy (Gemcitabine 1000 mg/m2 on days 1, 8, and 15; Cisplatin 70 mg/m2 on day 2; the cycle repeated after 30 days). On pre-chemotherapy blood investigations, her hemoglobin, total leukocyte count (TLC), platelet, urea, creatinine, sodium, and potassium were 7.5 g/dl, 7.75/μl, 1.45 lakhs, 29 mg/dl, 0.9 mg/dl, 137 meq/l, 4.6 meq/l, respectively. The third cycle of chemotherapy was delayed as he developed intractable vomiting and flu-like symptoms (nausea, muscle pain, weakness, headache, and fatigue) with myelosuppression (leukocytopenia and thrombocytopenia). After three cycles of chemotherapy, her hemoglobin, total leukocyte count (TLC), platelet, urea, creatinine, sodium, and potassium were 6.3 g/dl, 3.45/μl, 0.65 lakhs, 32 mg/dl, 1.4 mg/dl, 135 meq/l, 4.7 meq/l, respectively. Further chemotherapy could not be given due to the patient's poor general condition. The disease progressed, and he eventually developed pulmonary and liver metastasis. He chose no further treatment except palliation care. He succumbed to death around nine months after the diagnosis.

## Discussion

Sarcomatoid urothelial carcinoma is a rare neoplasm of the urinary bladder comprising 0.2-0.6 % of all histological types. It is a rare but aggressive malignancy of the urinary bladder. It is indistinguishable from sarcoma and is a high-grade biphasic neoplasm with both malignant epithelial and mesenchymal components. The etiology of such tumors remains unclear though radiation and intravesical cyclophosphamide instillation therapy are typical risk factors. 50-79 % of patients with SCC were current smokers. Many cases do not present with any risk factors, but the association has been noted with post-renal transplant and pancreas transplant into the bladder [2]. The mean age of patients is around 66 years, but a range from 41 to 96 years has been observed. Most patients (89.1%) are white, and the male-to-female ratio is 3:1. Kikuchi et al. suggested that the frequent location of these tumors in the trigone is evidence of an origin from the Wolffian body [5]. In recent studies, carcinosarcomas' most common site was the bladder's lateral wall. In this case, the tumor originated from the right lateral wall. Patients often present with symptoms similar to typical urothelial carcinoma, including hematuria, dysuria, and obstructive symptoms like frequency. Nodal and visceral metastases are usually present at diagnosis (>20 % of patients). This patient presented with hematuria and the passage of clots. He eventually developed pulmonary and liver metastasis.

Sarcomatoid carcinoma is developed from a common pluripotent progenitor cell with the potential for epithelial and mesenchymal differentiation. Sung et al. showed a considerable overlapping loss of heterozygosity between the sarcomatoid and carcinomatous components. The uniform, non-random X-chromosome inactivation is consistent with the hypothesis that it is monoclonal in origin [6]. Völker et al. further demonstrated considerably, but not complete, overlapping genetic alterations by comparative genomic hybridization of the sarcomatoid carcinomas [7]. By immunohistochemistry, epithelial elements react with cytokeratin, and epithelial membrane antigen (EMA), whereas stromal elements react with vimentin, desmin, myoglobin, and S-100. In this case, the tumor was GATA 3 and CK7 positive for epithelial and S100 for mesenchymal (chondrosarsomatous) components.

Squamous cell carcinoma comprises 2-5% of bladder malignancies, but it is a more aggressive, commonly invasive disease that generally presents at a more advanced stage. It is an epithelial neoplasm exclusively displaying histological features such as squamous pearls, intercellular bridges, and keratohyalin granules. It is favorable for squamous markers like CK 5/6, P63, p40, CK 14, and desmoglein 3. However, its concurrent association with chondrosarcoma is infrequent. Except for a single case report of high-grade urothelial carcinoma of the right ureter with squamous cell carcinoma and sarcomatoid carcinoma differentiation, no literature is available where both variants exist simultaneously in a case of sarcomatoid urothelial carcinoma [8].

Sarcomatoid urothelial carcinoma of the urinary bladder is very aggressive, and there is no standard treatment for this disease. A multimodal approach that includes definitive surgery, local radiation therapy, and chemotherapy is often used. Early detection and aggressive surgery are the strategies to be sought. Transurethral resection and partial cystectomy carry the risk of incomplete tumor resection. Radical cystectomy with pelvic lymphadenectomy appears to be the primary treatment for superficial and deeply invasive diseases [9]. The limited role of TURBT, followed by adjuvant intravesical chemotherapy with drugs like pirarubicin, is there in patients with early-stage sarcomatoid urothelial tumors [10]. Neoadjuvant chemoradiotherapy with radical cystectomy showed some benefit in recurrence-free survival, but no improvement was there in overall survival. There is a report of durable, complete, local, and distant remission of metastatic sarcomatoid carcinoma of the bladder obtained by systemic chemotherapy alone with gemcitabine and cisplatin [11]. In this case, the patient refused the surgical option of radical cystectomy with pelvic lymph node dissection. He took three cycles of gemcitabine and cisplatin. Unfortunately, he failed to show any response to chemotherapy.

It has an inferior prognosis, with a median overall survival of about 14 months. In a large retrospective study of 221 cases using the Surveillance, Epidemiology, and End Results (SEER) Program database, 35.8 % underwent radical cystectomy (RC), while 15.8 % received adjuvant therapy along with radical cystectomy. 1, 5, and 10-year cancer-specific survival rates were 53.9, 28.4, and 25.8%, respectively [12]. The prognosis is relatively better in patients who undergo early radical cystectomy, and the patient in discussion survived only nine months after the diagnosis.

Conclusions

Sarcomatoid (Chondroid) urothelial carcinoma with concurrent divergent squamous cell carcinoma differentiation of the urinary bladder is a sporadic tumor with an inferior prognosis. Pathological diagnosis and staging are crucial for proper classification and predicting survival. No standard treatment is available till now, but multimodality care with upfront radical surgery is the key to prolonging survival.

## Conclusions

Sarcomatoid (Chondroid) urothelial carcinoma with concurrent divergent squamous cell carcinoma differentiation of the urinary bladder is a sporadic tumor with an inferior prognosis. Pathological diagnosis and staging are crucial for proper classification and predicting survival. No standard treatment is available till now, but multimodality care with upfront radical surgery is the key to prolonging survival.
